# The Potential of Telemedicine to Improve Pediatric Concussion Care in Rural and Remote Communities in Canada

**DOI:** 10.3389/fneur.2019.00840

**Published:** 2019-08-02

**Authors:** Michael J. Ellis, Kelly Russell

**Affiliations:** ^1^Department of Surgery, University of Manitoba, Winnipeg, MB, Canada; ^2^Pediatrics and Child Health, University of Manitoba, Winnipeg, MB, Canada; ^3^Section of Neurosurgery, University of Manitoba, Winnipeg, MB, Canada; ^4^Children's Hospital Research Institute of Manitoba, Winnipeg, MB, Canada; ^5^Pan Am Concussion Program, Winnipeg, MB, Canada

**Keywords:** concussion, traumatic brain injury, telemedicine, teleneurology, network

## Abstract

Concussion is a form of mild traumatic brain injury that affects thousands of Canadian children and adolescents annually. Despite national efforts to harmonize the recognition and management of pediatric concussion in Canada, timely access to primary and specialized care following this injury remains a challenge for many patients especially those who live in rural and remote communities. To address similar challenges facing patients with stroke and other neurological disorders, physicians have begun to leverage advances in telemedicine to improve the delivery of specialized neurological care to those living in medically underserved regions. Preliminary studies suggest that telemedicine may be a safe and cost-effective approach to assist in the medical care of select patients with acute concussion and persistent post-concussion symptoms. Here we provide an overview of telemedicine, teleneurology, the principles of concussion assessment and management, as well as the current state of concussion care in Canada. Utilizing preliminary evidence from studies of telemedicine in concussion and experience from comprehensive systems of care for stroke, we outline steps that must be taken to evaluate the potential of telemedicine-based concussion networks to improve the care of pediatric concussion patients living in underserved rural and remote communities in Canada.

## Background

Concussion has emerged as an important public health issue among children and adolescents living in Canada. Over the past 10–15 years, an increasing number of youth are presenting to emergency departments and primary care providers with head injury and concussion, placing significant demands on the Canadian healthcare system (1, 2). With timely medical assessment and proper education and guidance, the majority of pediatric concussion patients will make a complete return to sport and school activities within 1–4 weeks (3). Children and adolescents who sustain head trauma and do not have timely access to proper medical care are at risk of concussions, more severe forms of traumatic brain injury (TBI) or other serious neurological conditions going unrecognized and untreated. They are also at risk of premature return to sports that can lead to additional injury resulting in more severe or prolonged symptoms, and in rare cases fatal or disabling brain injury (4). Although most pediatric concussion patients can be successfully managed by their primary care provider, certain patients including those with pre-existing conditions, those who develop persistent post-concussion symptoms, and athletes returning to high risk sports often benefit from referral to medically-supervised multi-disciplinary pediatric concussion clinics that have the personnel, expertise, and diagnostic resources to meet their complex needs (5, 6).

Unfortunately, timely access to primary and specialized healthcare is not universally available to all youth in Canada, especially those living in rural and remote communities who can face significant geographic, socio-economic and cultural barriers to accessing these services (7–13). Over the past two decades, telemedicine has emerged as important tool to help address disparities in healthcare access for patients living in medically underserviced regions. With an accumulating base of evidence to support the feasibility, safety, clinical utility, and cost-effectiveness of telemedicine in the management of acute stroke (14–20), use of this technology is now rapidly expanding to deliver care to patients with other neurological disorders including concussion (21–27).

Here, we provide an overview of telemedicine and teleneurology and outline the principles of concussion management and the current state of concussion care in Canada. Drawing from preliminary studies examining the use of telemedicine in concussion and well-established systems of care for stroke, we present a vision for telemedicine-based concussion networks to serve as an innovative approach to help optimize care of pediatric concussion patients living in rural and remote communities in Canada. We also discuss the limitations, barriers and future research directions that must be addressed to support wider adoption and implementation of telemedicine-based concussion care in Canada.

## Definitions

To examine the potential use of new or emerging technologies to improve healthcare delivery, it is important to have a clear understanding of definitions and terminology. Telemedicine was a term first coined in the 1970's, which literally translates to “healing at a distance” (28). The World Health Organization has defined telemedicine as “the delivery of health care services, where distance is a critical factor, by all health care professionals using information and communication technologies for the exchange of valid information for diagnosis, treatment and prevention of disease and injuries, research and evaluation, and for the continuing education of health care providers, all in the interests of advancing the health of individuals and their communities” (29). Telemedicine-based patient care can be provided synchronously and asynchronously. Synchronous or “real-time” care is commonly provided through in-person bidirectional audio-visual videoconferencing between a patient at an “originating” site and a healthcare provider at a “distant” site. Asynchronous or “store-and-forward” care is provided by the transmission of medical information (e.g., clinical data, diagnostic test results) to a remote provider. An example of asynchronous care is an eConsultation whereby a referring provider sends clinical data to a distant specialist who can provide recommendations, test interpretation or arrange an in-person consultation with the patient (30, 31). In addition to contributing to direct patient care, telemedicine-based technology can also be used to facilitate other healthcare activities including education, research and administrative functions (i.e., telehealth).

## Current Use of Telemedicine for Neurological Disorders

Recent advances in the use of telemedicine to improve care for patients with neurological disorders, termed “teleneurology,” have been driven primarily by an increasing worldwide burden of neurological disorders coupled with persistent deficiencies in access to specialized neurological care. The emerging potential of telemedicine to transform care for patients with neurological disorders living in medically underserved communities is best exemplified by its application to those with acute stroke. Two decades ago, Levine and Gorman (32) proposed that telemedicine or “telestroke” could be used to increase timely access to specialized neurological care and thrombolytic therapy for patients with acute stroke presenting to rural and remote hospitals that were not staffed by stroke neurologists. Following this seminal commentary, subsequent work has established a firm base of evidence supporting the feasibility, safety, efficacy and cost-effectiveness of telemedicine-based acute stroke management (19, 20, 33). Research has now demonstrated that clinical outcomes are comparable between remote hospitals serviced by telestroke and comprehensive stroke centers and that telemedicine is also helpful for identifying patients who could benefit from other therapies such as endovascular interventions or emergency neurosurgery (16). Moving beyond initial acute stroke management, telemedicine is now used to facilitate multi-disciplinary care including intensive care monitoring, secondary stroke prevention and rehabilitation (14, 17). Today, telestroke is a well-established component of care for comprehensive stroke centers that can now provide expanded clinical coverage to vast geographic territories through distributed or “hub-and-spoke” regional telestroke networks. Published position statements and expert reviews continue to establish and refine standards for telestroke programs and networks offering even greater opportunity to improve quality of care and patient outcomes as well as identify areas of future research and implementation (15, 18, 20, 34–37).

Building on this experience with stroke, teleneurology has expanded to assist with the care of patients with other neurological disorders such as dementia, headache, multiple sclerosis, spinal cord injury, cerebral palsy, epilepsy and movement disorders (21, 23, 26, 27). Preliminary work has demonstrated the feasibility of academic centers with expertise in pediatric neurology to establish telemedicine-based networks that provide specialized care for youth with epilepsy and headaches (38, 39). In addition, there is now a strong body of literature to support the use of telemedicine to provide consultative and therapeutic services to patients with mental health disorders (40–42).

Using coordinated provincial and territorial telehealth networks, a growing number of Canadians are now benefitting from improved access to sub-specialty experts via telemedicine-based services such as in-person videoconferencing and eConsultation (30, 43–45). These programs and networks provide the critical technology, expertise, and infrastructure to facilitate further expansion of telemedicine-based neurological care for patients living in medically underserved rural and remote communities throughout Canada.

## Current State of Concussion Care in Canada

Concussion is a condition that exists along a clinico-pathological spectrum of TBI and arises from the transmission of biomechanical forces to the brain leading to temporary alterations in neurological functioning (46). Injury mechanisms among children and adolescents can vary by age but often involve falls, sports, motor vehicle collisions or other accidents (47, 48). Although most pediatric patients with an acute concussion will make a complete neurological recovery within 1–4 weeks with proper education and guidance, ~30% of patients will develop persistent post-concussion symptoms and benefit from additional multi-disciplinary assessment and management (3, 48). Failure to undergo prompt medical assessment following head injury can result in a delay in the diagnosis of severe forms of TBI, spine injuries and serious neurological conditions leading to death or disability. Concussion patients who do not receive proper medical care are also at risk of returning to sports prematurely leading to recurrent injury, more severe or prolonged symptoms and rare but catastrophic brain injury resulting from second impact syndrome or malignant cerebral edema (4).

Over the past 15 years, there has been a significant increase in the number of Canadian youth seeking medical attention for concussion and head injury. A retrospective population-based study in Ontario observed a 4.4-fold increase in the number of emergency department and physician office visits for concussion from 2003 to 2013 among youth 5–18 years of age (2). Similarly, a report from the Canadian Institute for Health Information indicated that over 17,000 youth were evaluated for sport-related brain injuries in emergency departments in Ontario and Alberta in 2016–2017 representing an increase of 28% over the past 5 years (1). Although the factors contributing to this recent increase remains unclear, increased recognition and reporting of concussions as well as increased concern regarding the long-term effects of these injuries likely play an important role. Following implementation of a school-based concussion policy among Ontario schools in 2014, there was a 30% increase in the monthly rate of emergency department visits for concussion and a significant increase in the proportion of concussions reported to have occurred at school (49).

To address the public health concern of concussions in Canada, Parachute, the Public Health Agency of Canada and national sport stakeholders published the *Canadian Guideline on Concussion in Sport* that outlines a standardized clinical pathway and recommendations to help improve concussion education, recognition, and management for sport- and non-sport-related concussion among youth and adults (50). In addition, the Ontario Neurotrauma Foundations (ONF) has published guidelines that outline standards for concussion clinics and provide clinical recommendations for the management of adult and pediatric concussion patients (51–53). Lastly, some provincial governments have demonstrated interest in enacting youth concussion legislation that would mandate that all youth with a suspected concussion undergo medical assessment and medical clearance prior to returning to school and sport activities (54). In March 2018, Ontario became the first province in Canada to enact youth concussion legislation, termed Rowan's Law (55).

Despite the increasing burden of concussion among Canadian youth, there are emerging concerns that some patients are not receiving adequate medical care following these injuries. National and international guidelines recommend that youth with a suspected concussion undergo urgent medical assessment and obtain medical clearance prior to returning to full sport activities (46, 50) However, a retrospective population-based study in Ontario from 2003 to 2013 found that only one third of youth sought medical follow-up or clearance following an initial visit for concussion (56). Although the responsibility to provide medical assessment and clearance for youth with concussion generally falls upon primary care providers, surveys conducted among Canadian family medicine physicians, pediatricians and emergency department physicians demonstrate considerable knowledge gaps with a significant proportion failing to provide proper post-injury guidance and care (57–60).

To address these complex issues, Canada has experienced an explosion in “concussion clinics” that advertise specialized care to this patient population. Despite increasing access to concussion care in some regions of Canada, research suggests wide variability in the personnel and practices among these facilities many of which do not meet ONF standards for concussion clinics (53). In a study examining providers and clinics advertising specialized concussion care on the Internet, only 40% indicated the presence of an on-site medical doctor. Access to healthcare professionals with expertise in TBI (e.g., rehabilitation medicine physicians, neurosurgeons, neuropsychologists, neurologists) were found to be limited at the majority of sites with very few having access to the full complement of specialists needed to provide comprehensive concussion care (61). A notable proportion of clinics were also found to advertise services that were not supported by current empirical evidence (e.g., baseline testing) or were being offered without appropriate on-site expertise (e.g., neurocognitive testing without the presence of an on-site neuropsychologist).

Although access to high-quality primary and specialized concussion care is a concern for youth throughout Canada, it is even more limited for children and adolescents who live in rural, semi-isolated and isolated communities. Access to healthcare is an important social determinant of health and it is well-recognized that patients living in rural and especially remote northern communities in Canada, a significant proportion of who are Indigenous, face numerous geographic, socioeconomic and cultural barriers to accessing primary and specialized healthcare services (7–13). Factors such as travel distances, access to ground transportation, the high costs of air travel, lack of child care, inability to take time off work, communication barriers and previous negative interactions with the healthcare system can all contribute to disparities in healthcare utilization among people living in rural and remote communities. Primary care within many remote northern Canadian communities is provided by community health centers that are staffed primarily by nurses and supported by periodically visiting nurse practitioners and physicians. Lack of access to advanced diagnostic imaging as well as emergency and specialized services within rural and remote communities makes travel to major urban centers unavoidable for many patients thereby placing an enormous financial burden on patients and the healthcare system. For patients required to travel long distances by air or ground during the harsh winter months, abrupt changes in weather and seasonal road conditions due to storms, blizzards, or frigid temperatures can also prevent safe travel for extended periods thereby contributing to additional delays in receiving timely medical care (8).

In light of these challenges, the Canadian Academy of Sport and Exercise Medicine, the College of Family Physicians of Canada and the Canadian Medical Association has recently recommended that telemedicine and other virtual networks be explored as novel approaches to improve access to concussion care in Canada (62). However, to fully evaluate the potential of telemedicine to improve care for concussion patients in medically underserviced communities requires an in-depth appreciation of the fundamentals of head injury and concussion management.

## Medical Assessment and Management of Concussion

Patients who sustain physical trauma to the head or neck can experience non-specific neurological symptoms such as headache, dizziness, sensitivity to light and sound, fatigue, and difficulty remembering and concentrating. They can also present with red flags such as worsening or severe headaches, neck pain, diplopia, seizures, changes in mental status as well as numbness or weakness of the extremities. To provide a medical diagnosis of acute concussion or persistent post-concussion symptoms a clinician must rule out more severe forms of TBI (e.g., subdural hematoma), cervical spine injury, and medical conditions (e.g., stroke, migraine, demyelinating disease, neoplastic or infectious conditions) that can present with non-specific neurological symptoms including red flags. Accordingly, international and national guidelines recommend that all patients with a suspected concussion undergo a medical assessment (46, 50). To complete a comprehensive medical assessment requires a primary care provider to conduct a clinical history, perform a focused physical examination, as well as order and interpret diagnostic tests (e.g., computerized tomography, blood work). The clinical history should include demographic information as well as details related to the mechanism of injury, initial symptoms, as well as the presence of red flags. Pre-existing conditions that effect concussion recovery or management should be collected including a history of previous concussion/TBI, migraine headaches, epilepsy, learning and mood disorders (63). The nature and severity of post-concussion symptoms should also be assessed using a validated and age-appropriate symptom inventory such as those included in Sport Concussion Assessment Tool 5 (SCAT5) or Child SCAT5 (46). The physical examination of patients with a history of head and spine trauma should ideally include a comprehensive assessment of level of consciousness and mental status as well as cranial nerve, motor, sensory, reflex, cerebellar, balance and gait testing. Assessment of vestibulo-ocular functioning including testing of convergence, smooth pursuits, saccades, and vestibulo-ocular reflex should be completed (64). Patients presenting with episodic vertigo should undergo Dix-Hallpike or Supine Roll testing to screen for benign paroxysmal positional vertigo (BPPV). Lastly, assessment of the cervical spine including range of motion and palpation for central and paraspinal tenderness should be performed.

Over the years several standardized concussion assessment tools have been developed including the SCAT (65), Vestibular Oculomotor Screening Tool (VOMS) (66) and the King Devick (K-D) test (67). Although these tools have been shown to be helpful in assisting with screening of athletes with suspected sport-related concussion, they do not include all of the objective physical examination tests that are required to comprehensively assess neurological, cervical spine, and vestibulo-ocular functioning in patients presenting with suspected acute traumatic brain and spine injury or persistent post-concussion symptoms. Additionally, these tools are not sufficient when deciding if an athlete with a suspected concussion is safe to return to sport.

While a medical diagnosis of concussion can usually be confirmed based on the results of the clinical history and physical examination, supplemental tests can be helpful in certain instances. In rare cases where patients present with non-specific or transient concussion-like symptoms, computerized neurocognitive testing performed by a clinical neuropsychologist can be used to screen for objective deficits in cognitive functioning (68). Diagnostic imaging (including plain radiographs, computerized tomography, and magnetic resonance imaging) should be considered in patients with suspected structural brain or cervical spine injuries and should be directed by validated clinical decision-making rules where available (5, 69–72). Patients presenting with post-traumatic seizures should also undergo electroencephalography.

Once a medical diagnosis of concussion has been confirmed, patients should be provided with verbal and written education regarding the signs and symptoms of concussion, warning signs that should prompt a return for repeat medical assessment as well as guidance about how to make a gradual and safe return to school, work and sport-related activities. Athletes should be provided written documentation regarding what sport-related activities they are medically cleared to return to. Following an initial brief period of rest (24–48 h), student-athletes should be advised to follow their respective Return-to-School and sport-specific Return-to-Sport strategies and seek medical follow-up prior to returning to full contact practices and games where applicable (50). At present, there is no gold standard test to confirm physiological recovery following concussion. In general, patients should be considered clinically recovered when they are asymptomatic at rest (or have returned to their pre-injury neurological status in those with pre-existing conditions such migraine or mental health disorders), are tolerating full-time school, work, and physical exercise without symptoms and have a normal physical examination (5). Physicians should also ensure that athletes returning to sports have successfully completed stages 1–4 of their sport-specific Return-to-Sport strategy (non-contact practice) without any concussion-like symptoms prior to providing written medical clearance for them to return to full contact practices and games. In patients with pre-existing conditions (e.g., previous concussions, ADHD, mental health disorders) and those returning to sports with a risk of repeat head injury, additional supplemental tests such as graded aerobic treadmill testing and neuropsychological testing can be helpful to assist with confirming clinical recovery (73, 74).

Emerging evidence suggests that persistent post-concussion symptoms are mediated by heterogeneous and often overlapping pathophysiological and psychological factors that can lead to exercise intolerance, vestibulo-ocular or cervical spine dysfunction, cognitive impairments, post-traumatic headaches and mental health disorders (75, 76). Patients with persistent post-concussion symptoms should ideally be referred to multi-disciplinary concussion clinics and programs that have on-site access to physicians with expertise in concussion and TBI and who work closely with licensed experts in disciplines such as neuropsychology, neurology, physiotherapy, exercise science, neuro-ophthalmology, and psychiatry (5, 50, 53). Together, these teams can facilitate additional testing, develop individually-tailored targeted rehabilitation programs to address the patient's persistent symptoms and facilitate multi-disciplinary clearance to return to full school, work and sport activities.

## Current Status of Telemedicine in Concussion Management

The preceding discussion on the principles of concussion management and access to concussion care in Canada provide important background to help evaluate the few clinical studies that have examined the use of telemedicine to assist with assessment and management of patients with concussion.

Among these studies, Vargas et al. (24) presented a case report of a 15 year old male who sustained a head injury during soccer and underwent initial medical assessment and normal computerized tomography of the brain at a local emergency department. Two weeks post-injury, the patient underwent consultation by a neurologist via real-time videoconferencing. Clinical history was collected and a physical examination was performed including assessment of cranial nerve and sensory functioning, muscle stretch reflexes, coordination, and gait (the use of a remote examiner or telepresenter was not indicated). The SCAT-2 was used to assess cognition and balance. Scores on the Headache Impact Test-6, Generalized Anxiety Disorder 7-item Questionnaire and Patient Health Questionnaire were reviewed as well as the results from previous computerized neurocognitive testing. Images from the patient's previous CT scan were also reviewed via a remote Picture Archiving and Communication System (PACS) system. Based on the patient's symptom burden and the results of the physical examination and computerized neurocognitive testing, the patient was advised to refrain from further physical activity pending an in-person consultation with a concussion specialist. The authors suggested that telemedicine was useful in identifying a concussed athlete and determining the need for follow-up assessment to inform additional workup and return-to-sport decision-making. In another study, Vargas et al. (25) examined the use of telemedicine to assist with the sideline assessment of collegiate football players with suspected acute sport-related concussion. A prospective cohort of 11 athletes with suspected concussion underwent assessment by a remote neurologist via real-time videoconferencing as well as in-person assessment by a sideline healthcare provider using the Standardized Assessment of Concussion (SAC), K-D Test and modified Balance Error Scoring System (mBESS). The examiners were blinded to each other's examination and return to sport decisions until the end of the assessment. A high level of agreement was found between in-person and telemedicine-based assessments for the SAC (100%), K-D test (assessments were within a difference of 3 s 100% of the time) and the mBESS (assessment were within 3 points 100% of the time). Furthermore, return-to-sport decisions between providers were in agreement 100% of the time. The authors concluded that sideline telemedicine evaluations were safe and effective in determining return to sport status in athletes with suspected concussion. Lastly, the present authors conducted a retrospective review of a pilot telemedicine program established between a provincial multi-disciplinary pediatric concussion program and a remote hospital in northern Manitoba, Canada located ~760 km away (22). Due to the limitations of performing a complete physical examination via unassisted videoconferencing, all eligible patients were required to have undergone a medical assessment by a physician or nurse practitioner prior to referral and were screened by a single neurosurgeon to determine whether initial assessment via real-time videoconferencing was feasible or whether an in-person assessment was warranted. During the study period, 20 patients (median age 13 years; range 1.8–17) were evaluated through the telemedicine program including 18 (90%) who underwent initial assessment via real-time videoconferencing. The median time from the date the referral was received and reviewed at the concussion program to the date of initial consultation with the neurosurgeon was 2 days. One patient who presented with transient monocular visual disturbance and neuroimaging evidence of an orbital floor fracture following an assault and one patient who was referred from a local emergency department but lived in another remote community underwent in-person initial assessment. Initial assessments using real-time videoconferencing consisted of a clinical history, administration of the Post-Concussion Symptom Scale which was faxed between sites, review of previous diagnostic imaging studies via a secure PACS system and an abbreviated physical examination performed without a remote examiner that included testing of gross extraocular movements, facial symmetry, tongue movement, pronator drift, cervical spine range of motion, symptom provocation during saccade and gaze stabilization testing, balance testing and immediate and delayed 5-word recall. Among this cohort, 17 patients were diagnosed with acute concussions, one patient was diagnosed persistent post-concussion symptoms and post-traumatic migraine headaches and two infants were diagnosed with head injuries. Patients were managed according to national and international guidelines and were faxed completed copies of the *Canadian Guideline on Concussion in Sport* Medical Assessment and Clearance forms to facilitate a gradual return to school and sport-related activities where applicable. Four patients were referred on for further assessment by other members of the multi-disciplinary team including a headache neurologist (2 patients), vestibular physiotherapist (1 patient), neuro-ophthalmologist (2 patients), pediatric ophthalmologist (1 patient), plastic surgeon (1 patient), exercise physiologist for graded aerobic treadmill testing (1 patient), mobile crisis and adolescent psychiatrist (1 patient). One patient was arranged an MRI of the brain. At the end of the study period, 90% of patients met the criteria for clinical recovery, one remained in treatment and one was discharged to the care of a headache neurologist. Overall, 80% of patients were managed exclusively by telemedicine. The estimated cost avoidance associated with the 66 telemedicine encounters (57 videoconferencing appointments and 9 telephone follow-ups) conducted for this cohort based on regional health authority road travel reimbursement rates was $40, 972.94 or $2048.65 per patient. Delayed follow-up (>1 month) among 16 patients who achieved clinical recovery revealed that no patient experienced recurrent symptoms or a new concussion following discharge from the telemedicine program.

Taken together, preliminary research suggests that the use of telemedicine to assist in the assessment and management of select concussion patients is feasible and may be a useful approach to enhance timely access to safe and cost-effective sub-specialty care for those living in medically underserviced communities. However, there are several limitations and barriers that must be considered and overcome to help optimize its use and adoption throughout Canada.

## Limitations and Barriers

From a clinical perspective, the most important limitation of using telemedicine to assist with the management of concussion patients is the inability to perform a comprehensive physical examination. As stated above, the physical examination is an essential component of the initial medical assessment of patients presenting with head trauma, allowing the physician to rule out more serious forms of TBI, cervical spine injury, and neurological disorders that can present with concussion-like symptoms. Subtle findings on the focused vestibulo-ocular and cervical spine examination can also be helpful in directing treatment of patients with persistent post-concussion symptoms. Although preliminary research suggests that certain aspects of the SCAT can be reliably assessed via in-person videoconferencing (25), complete assessment of cranial nerve (including fundoscopy), motor and sensory functioning, tone and reflexes, as well as assessment of the scalp, jaw and cervical spine cannot be accomplished unless another examiner or telepresenter is available to conduct these tests and present the results to the distant physician (26, 27, 77). Assessment of oculomotor and vestibular functioning including objective testing of convergence, saccades, and vestibulo-ocular reflex requires additional training and experience and therefore is unlikely to be reliably enhanced by use of an inexperienced telepresenter. Despite these limitations, research demonstrates that a small proportion of acute pediatric concussion patients (~30%) present with objective evidence of vestibulo-ocular and cervical spine dysfunction and that these abnormalities typically resolve with conservative management and without the need for any further diagnostic imaging or therapeutic intervention (78–81). Furthermore, rare patients who present with other post-traumatic co-morbidities (i.e., cranial neuropathies, brachial plexus traction injuries, central cord neuropraxia, BPPV) typically report clinical red flags or symptoms that alert physicians to these conditions. Lastly, those patients who present with a normal physical examination at initial assessment are unlikely to develop new physical examination abnormalities during longitudinal follow-up. Given these present limitations and clinical nuances, the use of telemedicine to assist in the management of concussion patients should be used selectively at this time and only in cases where there are clear geographic and logistical obstacles to providing in-person longitudinal assessment and care. Although the limitations of completing a comprehensive physical examination via telemedicine may be overcome in the future by more experienced telepresenters and new portable technology capable of assessing certain aspects of neurological functioning (e.g., oculomotor and vestibular function), physicians must always exercise a low threshold for requesting an in-person assessment whenever a more comprehensive physical examination, supplemental testing (diagnostic imaging, neuropsychological testing, graded aerobic exercise testing) or multi-disciplinary referrals are needed to optimize patient care. Table 1 provides preliminary clinical recommendations regarding the potential use of telemedicine in pediatric concussion patients in Canada.

**Table 1:** Preliminary recommendations regarding the use of telemedicine in pediatric concussion in Canada.

*Recommendations for primary care providers*
Primary care providers including physicians, nurse practitioners and nurses should consider referring pediatric concussion patients to multi-disciplinary pediatric concussion clinics or programs whenever it is felt the patient's needs cannot be adequately addressed by the primary care provider and their existing local resources.
Examples:
- Instances where the primary care provider does not feel qualified to complete a comprehensive medical assessment or provide medical clearance for the patient to return to sports or other activities - Patients for whom longitudinal medical follow-up is unavailable - Patients who present with signs or symptoms (e.g., seizures, focal neurological deficits) that may indicate a more serious brain or spine injury or other neurological disorder - Patients who may require diagnostic tests (e.g., imaging) that are not available near the patient's home community - Patients who develop persistent post-concussion symptoms (>1 month post-injury) - Patients with pre-existing conditions (e.g., migraine headaches, mood disorders) that can make it difficult to assess clinical recovery - Patients who experience an exacerbation of pre-existing conditions (e.g., migraine headaches, mood disorders) and may require multi-disciplinary care - Patients returning to contact or collision sports or other activities with an elevated risk of head injury - Patients with a history of multiple concussions, persistent post-concussion symptoms, or abnormal diagnostic imaging findings that require multi-disciplinary return to sport and sport retirement guidance
*Recommendations for physicians providing medical assessment and follow-up of pediatric concussion patients*
Telemedicine (e.g., real-time videoconferencing) may be considered for follow-up appointments in patients presenting with acute concussion or persistent post-concussion symptoms who live in rural and remote communities and have undergone in-person medical assessment by the treating physician. Telemedicine may be carefully considered to assist in the initial medical assessment and follow-up of patients with acute concussion who: live in rural and remote communities where medical follow-up is unavailable and for whom travel to a multi-disciplinary concussion clinic is difficult have undergone a previous medical assessment by a physician or nurse practitioner and have a normal physical examination do not report any signs or symptoms suggestive of a more serious brain or spine injury or other neurological disorder (e.g., focal neurological deficits, neck pain).
Physicians must maintain a low threshold for requesting an in-person assessment in instances where a patient is seen through telemedicine but a comprehensive physical examination, supplemental testing (diagnostic imaging, neuropsychological testing, graded aerobic exercise testing) and/or multi-disciplinary referrals are required to optimize patient care.
*Recommendations for other multi-disciplinary healthcare professionals*
Telemedicine may be considered by neuropsychologists, occupational therapists and psychiatrists to assist in the assessment and longitudinal care of concussion patients who develop persistent cognitive and mood-related symptoms. Telemedicine may be considered by headache neurologists to monitor concussion patients with persistent headaches and who have undergone previous in-person assessment. Telemedicine may be considered by vestibular and cervical spine physiotherapists as well as exercise physiologists to advance treatment plans in patients who have undergone previous in-person assessment.
Expert consensus and additional research will be needed to refine these recommendations in the future.

In addition to these clinical limitations, there are a number of other important patient, technological, economic, medico-legal, and administrative barriers that must also be considered and addressed (82). Despite increasing access to specialized care and reducing patient travel, there is concern that the use of telemedicine may alter the patient-physician relationship. Trust, rapport and clear communication between patients and providers are important keys to effective healthcare and may be more difficult to establish via telemedicine vs. in-person encounters. Additional research is therefore needed to assess patient, parent, and healthcare provider experience, comfort and satisfaction with concussion telemedicine programs and to identify ways these services can be optimized to provide user-friendly, culturally-appropriate patient-centered care.

The potential of telemedicine to bridge gaps in concussion care in Canada is also critically dependent on a number of technological factors that facilitate access to reliable, high quality in-person videoconferencing. The most common systems used include mobile carts or wall-mounted cameras that can be controlled by the distant physician who connects via a secure PC-based software program that meets institutional personal health information and privacy requirements. However, some remote and northern communities may not have access to sufficient bandwidth to support high quality videoconferencing thereby restricting the expansion of telemedicine care to these regions and requiring patients to travel to other communities with these capabilities.

Establishing novel telemedicine sites, even within established telemedicine networks, can be associated with significant upfront capital costs for equipment, credentialing, and ongoing technical support and maintenance. These initial costs are often borne by institutions and healthcare providers while the long-term cost savings of telemedicine programs often benefit the healthcare system and society in general (83). As with other networks and programs, the future growth of telemedicine in concussion will require evidence that these approaches can provide reliable, efficient and cost-effective care. Lastly, there are important medico-legal and administrative barriers for those physicians and healthcare professionals providing patient care through telemedicine. Without a national framework for telemedicine, the Canadian Medical Protective Agency recommends that physicians providing telemedicine-based care consult their provincial or territorial colleges, some of which have published bylaws and polices (84). For those who provide care to patients across provincial and territorial borders, physicians are currently required to become licensed within the jurisdictions in which they and their patient reside. Although there is growing support for adopting a national license for physicians, licensing requirements currently vary across provinces and territories and may be an administrative and financial obstacle for some sub-specialty physicians who wish to offer telemedicine services to patients across their vast institutional catchment areas. Physician reimbursement for telemedicine-based services also varies across Canadian provinces and territories. The development of billing tariffs that provide remuneration for videoconferencing assessments and telephone follow-ups that are equivalent to that which is earned for in-person visits will be essential to increasing the number of physicians who are willing to add this service to their clinical practice model. In order to decrease physician medico-legal risk and liability, additional research will be needed not only to identify ways that telemedicine may improve concussion care but also ways in which it can be potentially harmful for select patients. Most recently, the Canadian Medical Association, Royal College of Physicians and Surgeons of Canada and the College of Family Physicians of Canada announced the development of a new national taskforce on virtual care that will hopefully address many of these outstanding challenges (85).

## Future Directions

**Figure 1:**
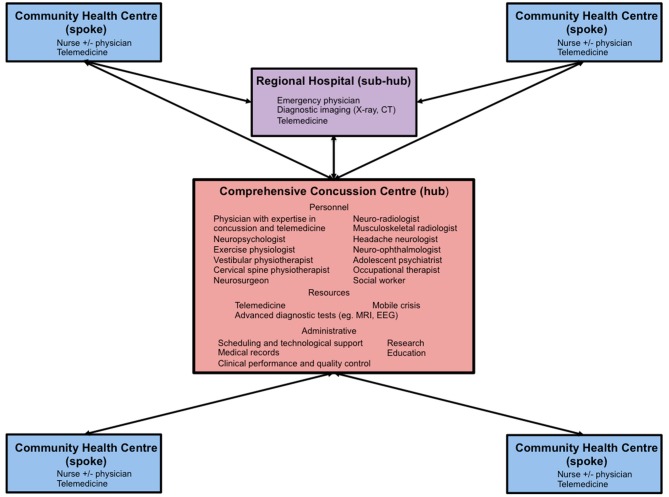
Diagram of a proposed “hub-and-spoke” regional pediatric concussion network. CT, computerized tomography; MRI, magnetic resonance imaging; EEG, electroencephalography.

Given the increasing burden of concussion and mild TBI among Canadian children and adolescents as well as the challenges of accessing specialized medical care among those living in rural and remote regions, it is incumbent on leaders in healthcare to develop innovative and sustainable strategies to address this public health issue and prevent its adverse short and long-term consequences. By overcoming many practical limitations and barriers, telemedicine has emerged as an important platform that can level the playing field and bridge the gaps in care for patients with neurological disorders living in medically underserviced communities. Although preliminary evidence suggests that telemedicine can be used to assist with the sideline assessment of athletes with suspected sport-related concussion (25), this application will likely be of limited use in Canada where national guidelines recommend that all youth with a suspected concussion undergo immediate and permanent removal from their game or practice and be referred for urgent medical assessment by a physician or nurse practitioner regardless of the results of sideline assessment (50). However, by following a roadmap outlined by telestroke, we believe the power of telemedicine can be harnessed to similarly transform and optimize the post-injury medical care of pediatric concussion patients in Canada. Similar to stroke, the model of care that has the greatest potential to be successfully applied to concussion in Canada is the “hub-and-spoke” model. In this system of care, a comprehensive stroke center that meets strict credentialing guidelines serves as the “hub” and provides clinical coverage and consultative services across multiple rural or remote hospitals or “spokes” that do not have access to similar multi-disciplinary expertise and diagnostic resources (16, 17). In more complex systems of care, hospitals that have access to some but not all of the personnel and resources of a comprehensive stroke center act as intermediate sites or “sub-hubs.” The authors envision a similar model of care for pediatric (and adult) concussion patients in Canada whereby credentialed university-based regional or provincial comprehensive concussion centers that have on-site access to physicians with expertise in concussion, a dedicated team of multi-disciplinary experts with licensed training in TBI sub-disciplines and appropriate diagnostic and administrative resources would serve as hubs and provide integrated care to healthcare centers and providers across broad geographic catchment areas using existing provincial and territorial telemedicine networks (see Figure 1). Patient care would be guided by current national guidelines (Parachute, Ontario Neurotrauma Foundation) and facilitated by the use of national harmonized resources such as the *Canadian Guideline Concussion in Sport* Medical Assessment and Clearance Forms and additional Post-Concussion Education Sheets that are currently being adapted for First Nations, Metis and Inuit youth. Given that many university-based pediatric concussion clinics are frequently involved in research and are linked through national research and clinical guideline working groups (Canadian Traumatic Brain Injury Consortium, Parachute, Ontario Neurotrauma Foundation), these centers and networks would be in an optimal position to conduct collaborative multi-institutional research including randomized controlled trials and cost utility studies to establish a firm base of evidence to support further implementation of these approaches and develop consensus standards and guidelines that would ensure quality control and help secure sustainable funding for these programs. In addition to care for patients with concussion and mild TBI, these networks could also support the multi-disciplinary care of pediatric moderate and severe TBI patients for whom travel is even more challenging due to difficulties with mobility and who require ongoing specialized neuro-physiotherapy, occupational therapy, speech language pathology and neuropsychological services (86) that are unavailable within rural and remote communities.

Driven by a mandate to provide timely access to comprehensive concussion care throughout Manitoba as well as government interest in establishing province-wide youth concussion legislation, the Pan Am Concussion Program in Winnipeg, Manitoba has now established Canada's first provincial pediatric concussion telemedicine program. The Pan Am Concussion Program is a provincial government-funded clinical program that accepts referrals for pediatric sport- and non-sport related concussion patients as well as youth with more severe TBIs who require additional care and rehabilitation. The program serves a geographically and culturally diverse catchment area that includes the province of Manitoba, eastern Saskatchewan, northwestern Ontario and central Nunavut. At this facility, all patients undergo medical assessment and clearance by a neurosurgeon who works with other multi-disciplinary team members including licensed experts in neuropsychology, vestibular and cervical spine physiotherapy, exercise science, neuro-ophthalmology, psychiatry, neurology, and others. Based on encouraging results for a pilot study (22), the Pan Am Concussion has partnered with MBtelehealth to expand the Concussion in the North EConsultation and Telemedicine (CONNECT) Program that provides eConsultation and in-person videoconferencing services for patients who have undergone initial medical assessment and referral by primary care providers across Manitoba. All patients living in rural and remote northern communities who have undergone initial in-person medical assessment at the concussion program and who have a normal neurological examination are considered for longitudinal follow-up using telemedicine. Acute concussion patients living in remote or isolated northern communities who have undergone a prior medical assessment by a primary care provider are considered for initial medical assessment and follow-up via in-person videoconferencing as long as there are no clinical concerns for more severe forms of TBI, cervical spine injury or other co-morbidities. In all cases, the neurosurgeon exercises clinical judgment when selecting cases to be assessed and followed through telemedicine and maintains a low threshold to request an in-person assessment in instances where a more complete physical examination or additional testing is required to optimize patient care. For those who do require further advanced diagnostic tests or multi-disciplinary consultations, these appointments are coordinated by the concussion program in order to limit the number of trips away from home. As pointed out by others, the success and sustainability of telemedicine networks is highly dependent on the engagement of clinical champions across the hub and spoke sites (36). Accordingly, our pediatric telemedicine program has benefitted from close working relationships with local healthcare and telemedicine providers within rural and northern communities, organizations that provide physician coverage to these regions (Northern Health Region, Ongomiizwin Health Services), Health Canada's First Nations and Inuit Health Branch, Pan Am Clinic as well as our regional health authorities (Shared Health, Winnipeg Regional Health Authority) and provincial telemedicine provider (MB Telehealth). Moving forward, this program will serve as an important platform to further evaluate the potential of telemedicine to provide safe, timely, effective, equitable, efficient, cost-effective, and culturally-appropriate patient-centered care for pediatric concussion patients living in rural and remote communities in Canada.

## Conclusions

Concussion is common injury among Canadian youth that is placing an increasing burden on the healthcare system. Although timely medical care is paramount to confirming a medical diagnosis and instituting evidence-based recommendations and care, access to physicians with expertise in concussion remains a challenge for Canadian youth especially for those living in rural and remote communities. Accumulating evidence supports the use of telemedicine to improve access to specialized care for patients with a wide spectrum of neurological disorders. Although preliminary research suggests that telemedicine may be a safe and cost-effective approach to improve care of select concussion patients living in medically underserviced communities, future research is needed to overcome remaining limitations and barriers and build a firm base of evidence to support the development and sustainability of telemedicine-based concussion networks in Canada.

## Author Contributions

ME and KR: conception and design of the work, drafting the work and revising it critically for intellectual content, final approval of the version to be published, and agreement to be accountable for all aspects of the work ensuring that questions related to the accuracy and integrity of any part of the work are appropriately investigated and resolved.

### Conflict of Interest Statement

The authors declare that the research was conducted in the absence of any commercial or financial relationships that could be construed as a potential conflict of interest.
